# Development and Implementation of a Context-Specific Multi-modal Point-of-Care Ultrasound Curriculum for a Kenyan Family Medicine Residency Program

**DOI:** 10.7759/cureus.75655

**Published:** 2024-12-13

**Authors:** Luke R Bishop, Jonathan Swanson, Fridah Kiptui, Erin F Shufflebarger, James C Crosby, Matthew A Heimann, Christopher Greene, Ashton Kilgore, William R Davis, Katherine Griesmer, Christine Shaw, Dino Crognale, Matthew C Larrison, Samuel L Burleson

**Affiliations:** 1 Emergency Medicine, University of Alabama at Birmingham, Birmingham, USA; 2 Emergency Medicine, Tenwek Hospital, Bomet, KEN; 3 Family Medicine, Tenwek Hospital, Bomet, KEN; 4 Emergency Medicine, HCA Florida West Hospital, Pensacola, USA; 5 Radiology, University of Alabama at Birmingham, Birmingham, USA

**Keywords:** curriculum, kenya, low-resource setting, pocus, point-of-care ultrasound, post grad medical education

## Abstract

Access to diagnostic imaging is significantly limited in much of the world, and sub-Saharan Africa is no exception. Clinician-performed point-of-care ultrasound (POCUS) may provide increased access to diagnostic imaging for many patients in low-resource settings, but training in this modality is limited. We describe the development and implementation of a context-specific, multi-modal pilot POCUS curriculum involving hands-on instruction, in-person and online didactics, asynchronous online image review, and quantitative evaluation. The curriculum was specifically designed for family medicine residents at a rural Kenyan training hospital.

This evidence-based training curriculum was designed for integration into a residency curriculum to train Kenyan family medicine physicians to achieve competence in POCUS use and develop the local expertise and leadership necessary to reproduce the training at other institutions. The curriculum was designed specifically for this Kenyan context; however, we provide a detailed description of all curricular elements and review the evidence informing those elements in order to facilitate reproduction at other similar institutions and settings to improve access to POCUS training.

We trained eight family medicine resident participants, all of whom strongly agreed with the utility of the curriculum and its component parts. All eight trainees met quantitative competency measures by written evaluations, direct observation, structured clinical exams, image review, and overall numbers of POCUS exams. A total of 1029 ultrasound scans were performed by the participants in the first year of implementation, averaging 128 scans per participant. No participant fully completed the required number of 165 scans for each application; however, most participants are continuing to add to their numbers as planned. Many of these scans were performed under direct faculty supervision to allow for real-time assessment and feedback, and the rest were asynchronously reviewed. All participants also passed all five observed standardized clinical evaluations (OSCEs), demonstrating their competency to perform, record, and interpret images in a timely and accurate manner. We describe many of the logistical requirements and challenges we experienced, as well as our methods of adapting to or overcoming them.

Our curriculum is an effective means of developing POCUS competence in an African setting. Our data and experience with implementation may help establish or expand POCUS into medical training in other institutions in sub-Saharan Africa, improving access to this vital diagnostic tool.

## Introduction

In much of the developing world, diagnostic imaging remains limited. The World Health Organization estimates that two-thirds of the world’s population has no access to basic radiology services [1]. Even if X-rays or computed tomography (CT) were readily available and affordable, there are far too few practitioners trained in image acquisition and interpretation. For example, Kenya, a country of 43 million, has only 200 radiologists [2]. Clinician-performed point-of-care ultrasound (POCUS) takes on a new level of relevance in these settings, as there is often no access to confirmatory studies.

POCUS has been recognized as one promising solution to the lack of diagnostic imaging in resource-limited settings (RLS) [3]. POCUS has long been utilized in emergency departments and intensive care units of high-income countries to quickly diagnose critical pathology [4]. With smaller and more affordable ultrasound equipment now available, POCUS can be used by any trained clinician, anywhere in the world. The diagnostic utility of POCUS in RLS extends across a spectrum of medical pathologies and specialties, including cardio-pulmonary, abdominal, obstetrical, trauma, and tropical infectious diseases, among others [5].

POCUS use in RLS leads to changes in patient management in up to 70% of patients scanned [6-10], often changes in medication or disposition, but sometimes surgical plans as well. Diagnostic accuracy, particularly in cardiac pathology, is significantly increased with POCUS use in RLS [9,11,12] though this may not always translate to improved clinical outcomes [12,13].

International organizations, including the African Federation for Emergency Medicine (AFEM), have called for incorporating POCUS training into their healthcare systems and training [14]. Despite these initiatives, access to even basic ultrasound training in many of these settings remains limited. In surveys, almost one-third of all African healthcare practitioner respondents affiliated with the AFEM did not use POCUS in their clinical practice [15]. This and other surveys report an overwhelming majority stating their lack of POCUS use was due to a lack of ongoing training or mentorship [15,16].

Several POCUS training courses are available to healthcare providers in RLS [10,16,17], but many are short-term initiatives that rely, at least initially, on trainers from high-income countries. Short courses may be effective but may also leave participants without ongoing mentorship or quality review, without sufficient physical resources, or without sufficient expertise to teach other clinicians in their settings. Long-term reliance on international expert teachers may not be sustainable, given the significant monetary, time, and travel investments required, as well as the possibility of inhibiting the growth of local leadership and expertise. While there are some resources for developing long-term and self-sustaining solutions [18], in this report, we describe the POCUS program implemented into the existing longitudinal curriculum of a family medicine residency program at a referral center in southwest Kenya.

The purpose of this report is to describe in detail the components and initial implementation of our multi-modal pilot curriculum with the ultimate goal of establishing a stable, self-sustaining, locally administered, and clinically relevant POCUS program. We believe our curriculum is one model to improve access to emergent diagnostic imaging and alleviate the lack of sustainable POCUS training and mentorship in RLS, and that it could be reproduced at other training programs in sub-Saharan Africa.

## Materials and methods

Setting and equipment

Our curriculum was implemented at Tenwek Hospital, in Bomet, Kenya, a tertiary care regional hospital in the southwestern highlands of Kenya. This hospital provides outpatient, inpatient, and intensive care to adult, pediatric, and maternity patients, as well as general and specialty surgery. It is a regional referral hospital and provides advanced imaging modalities (radiography, ultrasound, endoscopy, and CT), remote radiology coverage, and is currently building a cardiothoracic surgical center. It also provides a number of specialty services through its outpatient clinics, including cardiology, cardiac surgery, gastroenterology, comprehensive maternofetal medicine, ophthalmology, and primary care, including care of chronic tuberculosis and HIV infections. The hospital serves as the primary training site for residencies in orthopedics, general surgery, and family medicine, and a general medical internship for medical officers and clinical officers. Emergency and acute care constitutes a component of daily practice for family medicine trainees in this setting.

Given access to reliable radiology, POCUS in this setting is often a clinician-driven supplemental imaging modality used to assist in real-time clinical decision-making. However, many patients at the hospital and their families still struggle to access advanced imaging due to cost, machine maintenance, or radiologic interpretation. Additionally, after graduation, many trainees practice in settings without reliable access to traditional radiological imaging, making POCUS training during residency increasingly relevant. Hospital and residency program leadership recognized this need and requested the development of a POCUS curriculum.

For our initial implementation, we targeted all family medicine residents at the hospital. The hospital has reliable access to the internet, and all trainees have personal devices capable of accessing it. The institution acquired ten Butterfly iQ+ devices (iQ+, Butterfly Network, Inc., Burlington, MA, USA) that are available to clinical staff and trainees, in addition to a single traditional console-based machine (GE Venue Go, GE HealthCare Technologies Inc., Chicago, IL, USA). These were acquired through a combination of hospital budget and donation from third-party sources and were obtained prior to our curriculum development. As of this writing, an iQ+ is available for purchase for USD 2699, with a single yearly license of USD 420, which can support multiple devices. This is in contrast to traditional cart-based machines, which cost tens of thousands of dollars. Some discounts may be available for clinicians or institutions practicing in RLS. The iQ+ devices were kept by the heads of various clinical departments and stored on wards or in clinics but were accessible to all trainees by a “check out” system. All iQ+ devices were linked to the single hospital account for the Butterfly Cloud system (Butterfly Network, Inc., Burlington, MA, USA), while each participant had their own individually labeled portfolio archive within that cloud system for image storage and review.

Given the need for portability, affordability, and access in RLS, we chose to perform the majority of training with the handheld iQ+ devices. Access to POCUS devices after residency training will vary for individual graduates. However, we expect the trainees are more likely to have access to handheld ultrasound platforms for the performance of POCUS after leaving their current institution, given the lower cost, smaller size, and rapid proliferation of these devices. Some reports indicate handheld machines are adequate or non-inferior in RLS [5,19]. Therefore, we limited our core curriculum to exams that could be performed by a handheld ultrasound device.

Needs assessment

The potential discordance between traditional emergency POCUS curricula and the needs of RLS medical trainees in Africa has been called into question in previous reviews [18,20,21]. In order to avoid redundant or non-applicable POCUS training, we performed a targeted qualitative needs assessment with the residency director, hospital director of medical services, clinicians at the institution, and international volunteers with extensive experience in POCUS education and at the training institution to determine the most common clinical questions or applications to which POCUS could be useful and fit the resources of the institution. This working group represented a wide perspective of the pathology seen locally, the capabilities and resources available at the institution, and the scope of POCUS practice and literature.

This needs assessment revealed the POCUS curriculum should include many standard applications in emergency medicine and family medicine but also revealed the need for several expanded applications. Given the high prevalence of rheumatic heart disease and the availability of intervention at the institution, the curriculum included an expanded cardiac assessment [22,23]. Additionally, the prominent role of the learners in providing primary obstetric care and the high prevalence of both HIV and tuberculosis, expanded obstetric assessment [24] and the Focused Assessment with Sonography in HIV-Associated Tuberculosis (FASH) exam [7] were all included. Aorta exams are sometimes not included in POCUS curricula in RLS [21]; however, we chose to include the exam given its simplicity, its inclusion in the FASH exam, and the availability of cardiothoracic surgery at the Kenyan institution, rendering effective treatment possible.

To facilitate this type of an expanded ultrasound program, we formed an interdisciplinary curriculum task force with a radiologist, an obstetric sonographer, a primary care physician with formal echocardiographic training, and several emergency medicine physicians with extensive POCUS experience and with specific fellowship training in POCUS in RLS [25]. The team consists of trainers based primarily in the United States, as well as at the participating hospital, with the goal of incorporating Kenyan graduates of the curriculum into program leadership. This hybrid model addresses the well-described problem of maintaining mentorship and continuity through a training program [15,16].

Curriculum

Our curriculum and its implementation were reviewed and approved by both the Institutional Review Board of the University of Alabama at Birmingham and the Institutional Ethics Review Committee of Tenwek Hospital in Kenya.

The curriculum was created using principles modified from an established six-step approach for curriculum development [26]. This curriculum was designed to be implemented over the course of one year and repeated thereafter. Exams and applications were selected based on the needs assessment, by their inclusion in other current training curricula required for emergency and family physicians in the United States [27-29], evidentiary support [9-12,30,31], and applicability to East African RLS [7,22,23,32]. While our curriculum is organ-system-based, trainees were instructed throughout on the utility of combining various scans and applications into protocols to address particular clinical problems, such as the FASH exam or the combination of cardiac and lung exams in the evaluation of dyspnea or volume status. The curriculum is multi-modal, including asynchronous didactics, three large-group in-person sessions, and a monthly conference and image review. 

Our curriculum was initially implemented, and data was collected from January 1, 2023, to December 31, 2023. Prior to each in-person session, the learners completed asynchronous didactic modules tailored toward the specific exams to be taught that session. There are many excellent online POCUS resources available for such use. We chose POCUS 101 [33] for its step-by-step approach, brief individual modules, mobile phone app, low cost (USD 30 per learner with a group rate of up to 20 learners), and continuing medical education certification. Funding for the subscription was obtained through a third-party charitable organization chaired by one of the co-authors. Completion of these didactics prior to in-person sessions was monitored remotely by curriculum faculty through the POCUS 101 online application.

After completing the asynchronous modules, the learners participated in hands-on scanning sessions approximately every three months. Over the course of each one-week hands-on session, the learners participated in both in-person didactics reinforcing the asynchronous content and supervised ultrasound scanning of both standardized and real patients. Each session was held Monday through Friday, with approximately 30 hours of didactic time. Predetermined objectives and clinical questions for each exam (Table 1) were emphasized. These sessions were led by in-person trainers from the University of Alabama at Birmingham, USA, with extensive experience in POCUS and ultrasound education, with the plan to integrate and then transition to locally trained faculty in future years. A template schedule can be found in Table 2.

**Table 1:** Curriculum, objectives, and clinical questions for hands-on sessions AV: aortic valve; CRL: crown-rump length; EPSS: end point septal separation; LA: left atrium; LV: left ventricle; MV: mitral valve; OB: obstetrics; RA: right atrium; RHD: rheumatic heart disease; RV: right ventricle

Session	Exam or application	Clinical question	Specific criteria used, if applicable
1	Basics and Knobology		
	Cardiac [12,23,32]	IVC Collapse (>50%)?	
		Pericardial effusion/tamponade	Presence of effusion
			IVC collapse
			B-mode evaluation of RA and RV free walls
			M-mode comparison of RV free wall to MV in diastole
		LV ejection fraction by EPSS and visual estimation of wall motion?	High or hyperdynamic, normal, reduced, severely reduced
		Right heart strain?	RV>LV?
		Screen for RHD? [23]	LA>LV AV thickening
			MV regurgitation
			MV thickening
			Excessive MV anterior leaflet motion (hockey stick sign)
			AV thickening
	Lungs	Pleural effusions/hemothorax	
		Pneumothorax/lung sliding	
		A-lines v B-lines	
		Consolidations	
	Extended Focused Assessment with Sonography in Trauma (eFAST)	Hemopericardium	
		Hemoperitoneum	
		Hemothorax	
		Pneumothorax	
2	OB-Early	Confirmation of intrauterine pregnancy	Intrauterine gestational sac with at least one of the following: yolk sac, fetal pole, fetal heart tones
		Gestational Age	CRL
		Fetal heart tones by M-mode	
	OB-2^nd^/3^rd^ Trimester [24]	Fetal lie and presentation	Vertex?
		Number of fetuses	
		Placentation	Low-lying with 2cm of internal os or previa
		Fetal heart tones by M-mode	
		Amniotic fluid	Greatest vertical pocket amniotic fluid index if abnormal
		Fetal biometry	Biparietal diameter Abdominal circumference
			Femur length
			Head circumference
			Abdominal circumference
	Kidneys/bladder	Hydronephrosis	
		Ureteral jets	
		Bladder volume	
	Gall bladder	Gallstones?	
		Signs of cholecystitis	Pericholecystic fluid; gall bladder wall thickening; sonographic murphy’s sign
		Common bile duct dilation	
3	Lower extremity DVT	3-point compression	Saphenofemoral junction
			Femoral and deep femoral anastomosis
			Popliteal fossa
	Soft tissue	Cellulitis	
		Abscess	
	Aorta	Abdominal aortic aneurysm	
		Obvious dissection flap	
	Procedural	Peripheral vascular access	
		Needle guidance	
	Focused Assessment with Sonography in HIV-Associated Tuberculosis (FASH)	Ascites	
		Hepatic or splenic microabscesses	
		Pleural effusion	
		Pericardial effusion	
		Periaortic lymphadenopathy	
	Rapid Ultrasound for Shock and Hypotension (RUSH)	Cardiac as above	
		eFAST	
		Lung	
		Aorta	

**Table 2:** Sample week one schedule eFAST: extended Focused Assessment with Sonography in Trauma; RLS: resource-limited settings; OSCE: observed standardized clinical evaluation

Day	Time	Topic
1 - Monday		
	0830	Welcome, Orientation, Curriculum Overview, Schedule Overview
	0900	Lecture – Short Intro
	0930	Lecture – Intro to Cardiac US
	1030	Break
	1100	Hands-on Practice - Models
	1300	Lunch
	1400	Lecture – Intro to Thoracic
	1445	Hands-on Practice – Models
	1600	Finish
2 - Tuesday		
	0830	Intro to eFAST
	0930	Hands-on Practice - Models
	1030	Break
	1100	Scan in Emergency Department or Wards
	1300	Lunch
	1400	Scan in Emergency Department or Wards
	1600	Finish
3 - Wednesday		
	0830	Scan in Emergency Department or Wards
	1030	Break
	1100	Lecture – Cardiac US and Volume Assessment in RLS (Basic)
	1200	Lecture - Cases
	1300	Lunch
	1400	Scan in Emergency Department or Wards
	1600	Finish
4 - Thursday		
	0830	Scan in Emergency Department or Wards
	1030	Break
	1100	Lecture - Cases
	1230	Demonstration – Logging Scans for QA and practice OSCE
	1300	Lunch
	1400	Scan in Emergency Department or Wards
	1600	Finish
5 - Friday		
	0830	OSCEs – Cardiac and eFAST
	0930	Sonolympics
	1030	Break
	1100	Image Review (from the week)
	1300	Lunch and Finish

Assessments, competency, and image review

Competency in medical training and procedures is a complicated, difficult-to-measure entity, and POCUS competency is no different. Historically, documentation of a particular number of scans was deemed adequate for competency, and the American College of Emergency Physicians and American Association of Family Physicians recommend a minimum of 150 POCUS scans total (or approximately 25 scans per application) as a basis for competency [28,29]. Overall numbers are no doubt important, and increased numbers correlate with increased observed standardized clinical evaluation (OSCE) scores [34]. Current published POCUS expert guidelines [35] also delineate four sub-competencies in POCUS, using the acronym I-AIM, for Indications, Acquisition, Interpretation, and Medical decision-making. Our curriculum covered each of these sub-competencies for each application.

Learners were assessed in multiple ways for competency, including online written examinations, image review and feedback, OSCEs, and direct observation. Prior to each training session, each trainee was required to complete online exams via POCUS 101. During the weeklong training sessions, residents received real-time, in-person instruction, observation, and evaluation. At the end of each training week, trainees were evaluated on their scanning technique and image acquisition using OSCEs (Table 3) specifically developed for this program. OSCEs were administered for cardiac, extended Focused Assessment with Sonography in Trauma (eFAST), gallbladder, kidney/bladder, and the FASH exam with the addition of lung windows, all of which were selected due to their higher complexity level.

**Table 3:** Sample Observed Standardized Clinical Evaluation- Focused Assessment with Sonography in HIV-Associated Tuberculosis modified with lung windows

FASH OSCE			
Task	Performed w/o assistance (3 points)	Performed w/ assistance (1 Point)	Not performed (0 Points)
1. Greets patient, explains process, and explains free of charge			
2. Ensures proper patient positioning and privacy			
3. Turns on and positions machine appropriately (or verifies already done)			
4. Records patient data (to allow QA)			
5. Selects correct probe (curvilinear or phased arrays)			
6. Verifies probe is clean or cleans prior to use			
7. Appropriately adjusts depth			
8. Appropriately adjusts gain			
9. Obtains adequate view of Morrison’s pouch, revealing anterior liver tip			
10. Fans through liver parenchyma from Morrison’s pouch			
11. Records adequate view of Morrison’s pouch and liver parenchyma			
12. Obtains adequate view above R hemidiaphragm			
13. Records adequate view above R hemidiaphragm			
14. Obtains adequate view of R anterior superior lung windows			
15. Records adequate view of R anterior superior lung windows			
16. Obtains adequate view of L anterior superior lung windows			
17. Records adequate view of L anterior superior lung windows			
18. Obtains adequate view above L hemidiaphragm			
19. Obtains adequate view between L hemidiaphragm and spleen			
20. Records adequate view above and below L hemidiaphragm (may be same view)			
21. Obtains adequate view of spleno-renal space			
22. Fans through spleen parenchyma			
23. Records adequate view of spleno-renal space and spleen			
24. Obtains adequate subxiphoid cardiac view (parasternal long also acceptable)			
25. Records adequate subxiphoid cardiac view (parasternal long also acceptable)			
26. Obtains adequate short axis view of proximal abdominal (epigastric) aorta			
27. Records adequate short axis view of proximal abdominal (epigastric) aorta			
28. Scans down the aorta in short axis evaluating for peri-aortic LAD			
29. Records short axis scan down aorta			
30. Obtains adequate transverse view of suprapubic space			
31. Records adequate transverse view of suprapubic space			
32. Obtains adequate sagittal view of suprapubic space			
33. Records adequate sagittal view of suprapubic space			
34. Ends study			
35. Cleans patient			
36. Cleans probe			
37. Communicates findings and next steps to patient			
Score:__________/111 [>89 passing, 80%]			
Learner:_____________________________________________			
Examiner:____________________________________________			
Date:________________________________________________			

In between in-person training sessions, residents were required to perform and record scans. These exams were uploaded for online quality review. This review was done through the Butterfly Cloud system, led by curriculum faculty. Live remote imaging review conferences were held throughout the year in a group setting, often via a live Zoom session (Zoom Video Communications, San Jose, CA). These conferences were conducted roughly every other week, and all learners not otherwise occupied with their clinical responsibilities were strongly encouraged, though not required, to attend. These conferences were scheduled immediately after the learners’ core didactics to facilitate attendance.

Quantitative data used to track their progress included the number of ultrasound scans per application per participant, total POCUS scans per participant, and passed OSCE exams. Qualitative data included responses measuring how much their comfort grew in the use of POCUS, including performing the scans, interpreting them, and teaching them to other providers. Course faculty also evaluated participants qualitatively throughout each course.

Each trainee was expected to complete and document at least 25 of each type of exam, except for the skin and soft tissue, lower extremity deep venous thrombosis, aorta, and basic obstetric exams, of which 10 were completed. These exams were either performed with direct faculty supervision or were fully reviewed for quality purposes, and feedback was given by course instructors. Feedback was provided both in real time and by online Zoom image review and documented in the Butterfly cloud. If it was determined that the quality of the exams submitted was substandard, the trainee was asked to submit additional cases. Images acquired during in-person scanning sessions were often used in medical decision-making at the time of acquisition. Images obtained independently could also be used in clinical decision-making at the discretion of both the resident and attending physicians. Curriculum faculty were available directly by text message or email, though this was used very infrequently. One POCUS-fellowship-trained faculty member lived and worked full time at the training facility and was available for acute concerns.

Ultrasound-guided procedures did not have specific number objectives assigned to them, given the difficulty with high-fidelity simulation in this RLS and the irregularity of these procedures during hands-on training sessions. Instead, learners were specifically trained during hands-on workshops dedicated to ultrasound-guided needle placement and vascular access, and landmark and sonographic approaches were taught. Central venous catheterization was not included [21].

## Results

Eight family medicine residents in Kenya took part in the ultrasound training, and their progress was measured via quantitative and qualitative criteria. One emergency clinical officer participated in the first workshop, but due to clinical duties, could not complete the full curriculum and was excluded from further reporting. During the initial year of the curriculum implementation, a total of 1,029 ultrasound scans were performed by the eight family medicine resident participants, with an average of 128 scans per resident (range 90-155). Exams performed on standardized patients were not recorded but were counted toward a participant’s total number of scans on a manual log sheet. Exams performed under direct faculty supervision were counted towards a participant’s total and were recorded at the discretion of the faculty member at the bedside for future educational purposes or image review. All trainees passed all POCUS 101 modular quizzes with average scores greater than 90% and all five OSCEs with scores of greater than 80%, demonstrating basic knowledge and competency to perform, record, and interpret images in a timely manner. Residents were also continually assessed for competency during in-person scanning sessions and by asynchronous remote image review and were found to be competent and able to obtain and interpret scans appropriately. 

Seven out of eight participants responded to an anonymous survey at the end of the final session, and their answers were analyzed (Tables 4, 5). Participants rated statements on a one-to-five Likert scale, with one being “strongly disagree” and five being “strongly agree,” with scores of four and five being considered ‘favorable.’ Open-ended feedback was also collected, which was overwhelmingly positive, with many learners citing the positive impact this would have on their practice in the future. The only constructive criticism given in this section was requests to include even more types of scans in the future.

**Table 4:** Anonymous survey by participants evaluating various elements of the point-of-care ultrasound curriculum using the Likert scale 1 = strongly disagree; 5 = strongly agree; POCUS: point-of-care ultrasound

Survey question	Survey response after course
	n (%), Total = 7
This course improved my comfort with the use of POCUS in general	
Strongly Agree/Agree	7 (100)
Strongly Disagree/Disagree	0 (0)
After this course, I am more likely to use POCUS in my medical practice in the future	
Strongly Agree/Agree	7 (100)
Strongly Disagree/Disagree	0 (0)
I find the asynchronous modules helpful	
Strongly Agree/Agree	7 (100)
Strongly Disagree/Disagree	0 (0)
I find the in person lectures helpful	
Strongly Agree/Agree	7 (100)
Strongly Disagree/Disagree	0 (0)
I find the online image review sessions helpful	
Strongly Agree/Agree	7 (100)
Strongly Disagree/Disagree	0 (0)
I find the standardized patient scanning sessions helpful	
Strongly Agree/Agree	7 (100)
Strongly Disagree/Disagree	0 (0)
I find the patient scanning sessions on wards helpful	
Strongly Agree/Agree	7 (100)
Strongly Disagree/Disagree	0 (0)
After this course, I feel confident in my ability to teach other clinicians how to obtain adequate point-of-care ultrasound images	
Strongly Agree/Agree	5 (71.4)
Strongly Disagree/Disagree	0 (0)
After this course, I feel confident in my ability to teach other clinicians how to interpret point-of-care ultrasound images	
Strongly Agree/Agree	7 (100)
Strongly Disagree/Disagree	0 (0)

**Table 5:** Anonymous survey results regarding participants confidence in specific point-of-care ultrasound applications before and after the curriculum FAST: Focused Assessment with Sonography in Trauma; DVT: deep vein thrombosis; FASH: Focused Assessment with Sonography in HIV-Associated Tuberculosis

Survey question	Before course	After course
	n (%), Total =7	n (%), Total =7
I feel very confident in my use of point-of-care cardiac ultrasound in clinical practice		
Strongly Agree/Agree	0 (0)	6 (85.7)
Strongly Disagree/Disagree	7 (100)	(0)
I feel very confident in my use of point-of-care lung ultrasound in clinical practice		
Strongly Agree/Agree	0 (0)	7 (100)
Strongly Disagree/Disagree	7 (100)	0 (0)
I feel very confident in my use of the FAST exam in clinical practice		
Strongly Agree/Agree	0 (0)	6 (85.7)
Strongly Disagree/Disagree	5 (71.4)	0 (0)
I feel very confident in my use of point-of-care obstetric ultrasound in clinical practice		
Strongly Agree/Agree	0 (0)	6 (85.7)
Strongly Disagree/Disagree	6 (85.7)	0 (0)
I feel very confident in my use of kidney and bladder point-of-care ultrasound in clinical practice		
Strongly Agree/Agree	0 (0)	6 (85.7)
Strongly Disagree/Disagree	7 (100)	0 (0)
I feel very confident in my use of biliary/gallbladder point-of-care ultrasound in clinical practice		
Strongly Agree/Agree	0 (0)	6 (85.7)
Strongly Disagree/Disagree	7 (100)	1 (14.3)
I feel very confident in my use of DVT point-of-care ultrasound in clinical practice		
Strongly Agree/Agree	0 (0)	7 (100)
Strongly Disagree/Disagree	7 (100)	0 (0)
I feel very confident in my use of aorta point-of-care ultrasound in clinical practice		
Strongly Agree/Agree	0 (0)	5 (71.4)
Strongly Disagree/Disagree	7 (100)	0 (0)
I feel very confident in my ability to use point-of-care ultrasound for skin and soft tissue infections		
Strongly Agree/Agree	0 (0)	6 (85.7)
Strongly Disagree/Disagree	7 (100)	0 (0)
I feel very confident using the FASH exam in clinical practice		
Strongly Agree/Agree	0 (0)	7 (100)
Strongly Disagree/Disagree	7 (100)	0 (0)
I feel very confident in my use of ultrasound to obtain intravenous access		
Strongly Agree/Agree	0 (0)	4 (57.1)
Strongly Disagree/Disagree	7 (100)	0 (0)

## Discussion

Our curriculum is one model of a locally initiated and clinically relevant POCUS curriculum that establishes core competencies in POCUS for a family medicine residency program in rural Kenya. The curriculum was specifically created for general acute care clinicians in RLS in Kenya, but we believe the content and model are applicable in similar RLS contexts and could be generalized to other specialties with minor adaptations. It aims to invest heavily in a one-year, repeatable, longitudinal curriculum that integrates into existing educational and clinical infrastructure while still ensuring sufficient time for individual practice of scanning techniques, which bolsters long-term knowledge retention. Trainees and faculty who complete the program may also choose to repeat future iterations of the curriculum in order to develop further competence and expertise in POCUS applications. Trainees and local faculty exhibiting particular aptitudes and interests will also be progressively incorporated into the course leadership in future iterations of the curriculum to develop local leadership and sustainability.

Our curriculum was designed to be comprehensive while also still focusing on the organ-system-based modules on clinical integration and management. For example, our first session included the heart, lung, and FAST exams in order to facilitate the use of all exams in the evaluation of acute dyspnea, hypotension, or volume status. Both supervised scanning on the wards and cases presented during didactics focused on the clinical integration of these exams, rather than their separate use to evaluate a sole organ system.

Each component of the curriculum is critical and engages the learner through a different modality of knowledge acquisition and retention. A similar multi-modal fellowship training program in Peru has recently been described and successfully implemented by faculty in the United States [36]. Our three in-person sessions, while brief, allow for undistracted exposure to the material through formal didactics and bedside ultrasound scanning sessions. The specific elements of each session are listed in Table 1 and represent achievable, core elements that an engaged learner could expect to become proficient with after participation with this curriculum. The material is tailored to include enough core elements that no prior clinical ultrasound experience is required. While the initial target audience is resident physicians early in training, all local residents and faculty have the opportunity to participate until a sufficient cadre of proficient local faculty is available to teach further iterations of the curriculum. 

A strong asynchronous component is woven into the POCUS training curriculum. The online modules provide a low-cost, easily accessible, and repeatable method of transmitting foundational knowledge. Robust image review and quality assurance are indispensable for both trainee education and patient safety [18,28]; we accomplish this with online asynchronous image review. By utilizing proprietary cloud-based imaging programs, trainees are able to obtain feedback on their image acquisition and interpretation as well as track numbers. Furthermore, exposing learners to additional asynchronous education resources primes the students for more productive in-person sessions. These sessions were scheduled adjacent to other residency didactics to facilitate trainee attendance. We often had three or four of our eight trainees in attendance at a single session, depending on their clinical availability, though attendance was voluntary and not tracked. Reminders were sent through email or text. A single faculty member was charged with leading an individual session, but multiple faculty were often present for sessions. Leadership of sessions rotated between course faculty to minimize the time and scheduling burden. We found the group review session was most beneficial when the faculty member had already reviewed the images prior to the session, noting quality issues, teaching points, and interesting pathology beforehand. This allowed the group time to be more focused and efficient. Pre-session review took approximately one hour, with an additional hour for the session itself. Feedback was provided verbally in these sessions, as well as by comments in the imaging database. Group or individual emails and texts were also sent after sessions with reminders about image acquisition, interpretation, or documentation, particularly if common themes were noted during a session. Course faculty could not review images that were not properly recorded or labeled, but all recorded images were reviewed. Often multiple scan applications were indicated and done on a single patient and were counted separately towards a trainee’s total (i.e., cardiac, lung, and FAST on a single patient). We experienced few internet or video conferencing issues over the course of the curriculum.

All eight participants demonstrated competency and proficiency in multiple ways. All participants completed every asynchronous module prior to in-person courses and passed the written assessments included in those modules. All participants also passed each of the five OSCEs on the first attempt. 

We originally planned for participants to obtain 25 scans of each of the nine included modalities (Table 5), consistent with published policies for emergency physicians in training in the United States [28]. The curriculum was structured such that the more complicated exams were taught early in the curriculum to allow more time for practice and increased repetitions. These metrics and the timing of instruction within the curriculum were chosen intentionally to allow learners more time to practice the more complex scans requiring higher repetitions to master. More straightforward scanning applications were placed towards the end of the curriculum, where course faculty determined they could be adequately learned with fewer repetitions. For those included later, such as skin and soft tissue, aorta, early obstetric, and DVT exams, numbers were augmented with pathologic image review. Numbers for each of these applications were then lowered to 10 each, for a total of 165 scans. 

No participants were able to complete the proposed goal number of scans within the first year of implementation (165). However, we believe the fact that participants were able to complete an average of 128 scans per resident in less than a single year of POCUS training and have a 100% OSCE passage rate showed a clear level of POCUS competence. As a point of reference, an emergency medicine resident in the United States is required to perform a total of 150 POCUS scans over the period of a three-year residency program to be considered competent to use POCUS in clinical settings [27]. Additionally, our curriculum is designed to be implemented early in residency training, which will allow trainees ample time to continue to hone their skills and scan adequate numbers of patients to meet the numeric benchmarks. The only participants who were unable to continue scanning were those senior residents graduating at the end of the first year of implementation, and they received additional supervised scanning time with faculty after the conclusion of the final in-person course to gain additional experience. 

Participants strongly endorsed the inclusion of all curricular elements (Table 4), and we believe the quantitative findings of competency support the effectiveness of this curricular model. Survey results also revealed an increase in participants’ comfort in teaching POCUS to other clinicians (Table 4). However, as an ongoing effort to increase local expertise, leadership, autonomy, and sustainability of the curriculum, we are developing and implementing measures to develop program graduates and local faculty into local “POCUS champions,” who will develop both increased POCUS content mastery and teaching experience. These champions will be progressively incorporated into the planning and execution of the curriculum. Outcomes of these train-the-trainer measures are the subject of ongoing further investigation and are pending.

Based on formal and informal feedback from trainees and course faculty, we plan to make several changes in future iterations of the curriculum. These include an additional “intermediate” cardiac lecture, with more in-depth training in the evaluation of right heart strain, evaluation of pericardial tamponade, and rheumatic heart disease screening; shorter lectures overall to allow more hands-on scanning time while course faculty were present; more sessions focused strictly on sonographic pathology, particularly on pathology the trainees did not frequently encounter during the in-person weeks; and additional procedural guidance workshops.

Further avenues for expansion of the curriculum include the inclusion of clinical officers working in similar settings, physicians from other specialties, and physicians from other institutions traveling to the Kenyan institution for training. As local leadership develops and graduates and champions become competent and confident in their own ability to train other clinicians, the program could potentially be expanded to other institutions.

Limitations

This study demonstrates the implementation of a strong and potentially reproducible POCUS curriculum in an RLS; however, there are several limitations. The needs assessment was conducted at the implementing hospital in rural Kenya, and subsequently, the curriculum was tailored to these specific local needs. As a single-site needs assessment and subsequent single-site pilot implementation, generalizability to other locations may be limited. However, we believe this curriculum is relevant throughout sub-Saharan Africa, where similar disease prevalence exists.

There are challenges that must be acknowledged in implementing a longitudinal curriculum such as this. One barrier to sustainability is achieving an adequate number of POCUS-experienced faculty to lead and eventually multiply future training programs. This is a vital and ongoing effort as a part of our curriculum, but its outcome is yet to be determined at this early stage of implementation.

There is an appreciable investment in time and money to send United States-based faculty to lead the in-person large group sessions, with additional resources required to develop and train local POCUS champions. In our experience, a trip for a United States-based faculty member to travel to and from Kenya for an in-person course costs roughly USD 4000-4500, including airfare. This cost was mitigated for our team by the non-governmental organization that provides volunteer staffing for the Kenyan hospital, covering airfare for all volunteers. This reduced the cost by more than half, the rest of which was covered by professional reimbursement accounts provided by the university to faculty for professional academic pursuits. A single weeklong in-person training course, including travel, required as little as 10 days' absence from the United States. Each course required two or three faculty, with additional available help from Kenyan faculty.

Scheduling online didactic or image review sessions across eight time zones was challenging, despite the use of video conferencing. This was partially alleviated by scheduling these sessions adjacent to the residents’ pre-existing didactic commitments and assembling a team of international faculty who could alternate leading the sessions.

Time and space must also be allocated to the pre-existing residency educational curriculum. The removal of resident physicians from active patient care is a significant, possibly insurmountable, burden for many African hospitals. It is a measure of the local desire for and support of the POCUS curriculum that this accommodation was made at our institution. This burden may limit the wider generalizability of the current training model. However, we believe that this intensive investment will yield local expertise that can then lead to a more flexible model, when not dependent on international trainers. The curriculum could also be limited to a smaller portion of the residents in a given year so some could still be available for patient care. Potential avenues of further study include analysis of expansion, transfer to local train-the-trainers, sustainability, and possibly an advanced POCUS curriculum.

Beyond the curriculum and its implementation, many barriers exist to the clinical use of POCUS in RLS. These may include unpredictable power supply, internet access, machine cost and maintenance, and lack of supplies or other institutional support. These may be more or less pertinent in a particular institution. In our implementation site, institutional support was strong, with the hospital and program leadership initiating the request for the curriculum and already acquiring POCUS hardware prior to our implementation. Occasional iQ+ device maintenance issues arose but were often resolved by online contact with the manufacturer, troubleshooting with faculty, or bringing spare parts when traveling for hands-on courses. Other barriers may be overcome by handheld or battery-powered ultrasound machines, digital telecommunications, locally sourced supplies, and local ingenuity.

## Conclusions

We present a comprehensive evidence-based curriculum to address the shortage of self-sustaining POCUS training and mentorship which prevents its wider dissemination in low-resource settings. Our context-specific, multi-modal curriculum is initiated locally, driven by the pathology seen by acute care clinicians in Kenya, and takes advantage of advances in hand-held ultrasound technology, digital cloud-based storage and communication, and innovative asynchronous online POCUS education. Our participants displayed quantitative competence in POCUS use clinically and expressed significant increases in comfort level in all applications. We believe this model of curriculum is reproducible in other hospitals and training programs, especially as graduates develop local POCUS expertise and begin to sustain the program locally.
